# Cost‐Effectiveness of Treating Hepatitis C in Clients on Opioid Agonist Therapy in Community Pharmacies Compared to Primary Healthcare in Australia

**DOI:** 10.1111/jvh.14015

**Published:** 2024-10-23

**Authors:** Joshua F. Ginnane, Nick Scott, Andrew Radley, John F. Dillon, Margaret Hellard, Joseph Doyle

**Affiliations:** ^1^ Disease Elimination Program Burnet Institute Melbourne Victoria Australia; ^2^ School of Public Health and Preventive Medicine Monash University Melbourne Victoria Australia; ^3^ Division of Molecular and Clinical Medicine University of Dundee Dundee UK; ^4^ Directorate of Public Health, Kings Cross Hospital NHS Tayside Dundee UK; ^5^ Department of Gastroenterology, Ninewells Hospital and Medical School NHS Tayside Dundee UK; ^6^ Department of Infectious Diseases Alfred Health and Monash University Melbourne Victoria Australia; ^7^ Melbourne School of Population and Global Health University of Melbourne Melbourne Victoria Australia; ^8^ Doherty Institute University of Melbourne Melbourne Victoria Australia

**Keywords:** cost‐effectiveness analysis, hepatitis C, opiate substitution treatment, pharmacies

## Abstract

Meeting the World Health Organisation 2030 target of treating 80% of people with hepatitis C virus (HCV) in Australia requires accessible testing and treatment services for at‐risk populations. Previous clinical trials, including those in Australia, have demonstrated the efficacy of outreach programmes to community pharmacies offering opioid agonist therapy (OAT). This analysis evaluates the potential cost‐effectiveness of introducing an outreach programme in community pharmacies. Using a decision analytic model, we estimated the impact of adding a temporary hepatitis C outreach and treatment programme in community pharmacies to the standard treatment pathway available through general practice. We compared the expected number of tests, diagnoses, cures and costs occurring through the addition of this outreach and treatment programme to those expected through general practice alone over a 12‐month time horizon. We examined costs from the perspective of the health system and conducted one‐way and probabilistic sensitivity analyses to assess uncertainty in model parameters and test key assumptions. In the model adding the outreach programme pathway increased the number of tests from 4178 to 8737, the number of diagnoses from 615 to 1285 and the number of cures from 223 to 777 among people on OAT over a 12‐month period. Each additional cure achieved through the addition of the outreach programme was estimated to incur $48,964 (AUD 2023) to the health system, with > 85% of these costs attributable to medication and dispensing expenses. The average cost per cure was estimated to be $49,152 through routine care and $49,018 in the outreach programme. Although outreach models of care incur large upfront costs, they can capture otherwise unreached populations and result in comparable or favourable cost per cure, due to higher levels of engagement and lower rates of loss to follow‐up.

## 1 Introduction

Direct‐acting antiviral (DAA) medications for hepatitis C virus (HCV) have been widely available in Australia since 2016. Compared with earlier interferon‐based treatments, DAAs are highly tolerable and effective [1, 2]. Concerted efforts from specialist, general practitioners, non‐governmental organisations and public health authorities have resulted in more than 105,000 Australians receiving DAAs between 2016 and 2022 [3]. Both the incidence of new infections and prevalence of HCV in key populations—such as people who inject drugs, living with HIV and gay, bisexual and other men who have sex with men—are falling [4, 5]. Despite this, significant work will need to be undertaken to meet the target of eliminating HCV in Australia by 2030 [6].

Although HCV affects a diverse range of people across Australia, people who inject or have injected drugs remain a priority population for interventions in our national strategy [7]. People who inject drugs (PWID) experience a high degree of socioeconomic disadvantage, lower rates of education and higher rates of unemployment and unstable housing [8]. These concurrent challenges can result in the low prioritisation of seeking testing and treatment for HCV, especially when many people living with chronic HCV do not experience many symptoms and have limited knowledge of available treatment options [9, 10]. Those who do actively seek testing or treatment may face further challenges in the form of difficulty navigating services or through experiencing stigma [11]. Nevertheless, a large proportion of past or current PWID are engaged on OAT, which requires regular—sometimes daily—attendance at community pharmacies. In current models of HCV care in Australia, pharmacies are largely ignored and PWID or use of OAT pass through untested for HCV.

Models of care focusing on providing testing, counselling and treatment of HCV to patients on OAT through community pharmacies have been shown to be highly efficacious in randomised trials [12]. A real‐world, international, cluster randomised trial including 14 Australian sites (REACH‐HCV) reported that providing convenient testing and treatment through the pharmacy, where clients were already engaged on a weekly basis, resulted in a nine‐fold increase in cures [12]. This cost‐effectiveness assessment was undertaken to assess the potential clinical and financial impact of implementing a 12‐month HCV testing and treatment pathway in Australia based on the model demonstrated in REACH‐HCV which used nurses to deliver outreach services. We aimed to provide estimates of the number of HCV tests, diagnoses and cures that could be delivered through a pharmacy‐based model of care, as well as the incremental costs they would incur from the health system perspective compared to standard testing and treatment in primary healthcare.

## 2 Study Design and Methods

A cost‐effectiveness analysis using a decision analytic model was performed to assess the costs and outcomes of introducing a 12‐month nurse‐led HCV testing and treatment programme in community pharmacies in Australia, designed in line with the model of care demonstrated in the randomised intervention trial, REACH‐HCV [12]. A full description of the processes employed in REACH‐HCV is described elsewhere [12, 13]. Briefly, pharmacists dispensing OAT in suitable pharmacies were provided with training and literature to facilitate opportunistic discussions about HCV testing with OAT clients. If interested, OAT clients could then undertake testing with study nurses who performed venepuncture at the pharmacy. Test results were provided to participants by the study nurses over the telephone. People found to have HCV were then assessed for eligibility for treatment with DAAs. Treatment was prescribed remotely by medical staff in the Australian study sites and dispensed through the participants regular pharmacy. The intervention was compared to pharmacists referring OAT clients for testing and treatment with their usual general practitioner. The intervention was highly effective with participants far more likely to complete testing (OR 16.95, 95% CI 7.07–40.64) and be cured (OR 8.64, 95% CI 1.82–40.91) than in the conventional testing and treatment pathway [12].

The cost‐effectiveness analysis was undertaken from the health system perspective. The hypothetical model population consisted of individuals taking OAT dispensed through community pharmacy locations in Australia. The intervention assessed was the addition of the nurse‐led outreach pathway described above alongside existing testing and treatment pathways. The comparator was the expected testing and treatment of the same population through existing primary healthcare pathways. The primary outcome was to assess the incremental cost‐effectiveness ratio, meaning the incremental cost incurred per incremental HCV cure gained with the addition of the outreach programme in Australian dollars (AUD, 2023). No cost‐effectiveness threshold was set. Secondary outcomes were the number of expected tests and diagnoses the programme could deliver in a one‐year period. As health outcomes and costs were calculated over a 12‐month time horizon, discounting was not applied.

## 3 Model Structure

**FIGURE 1:**
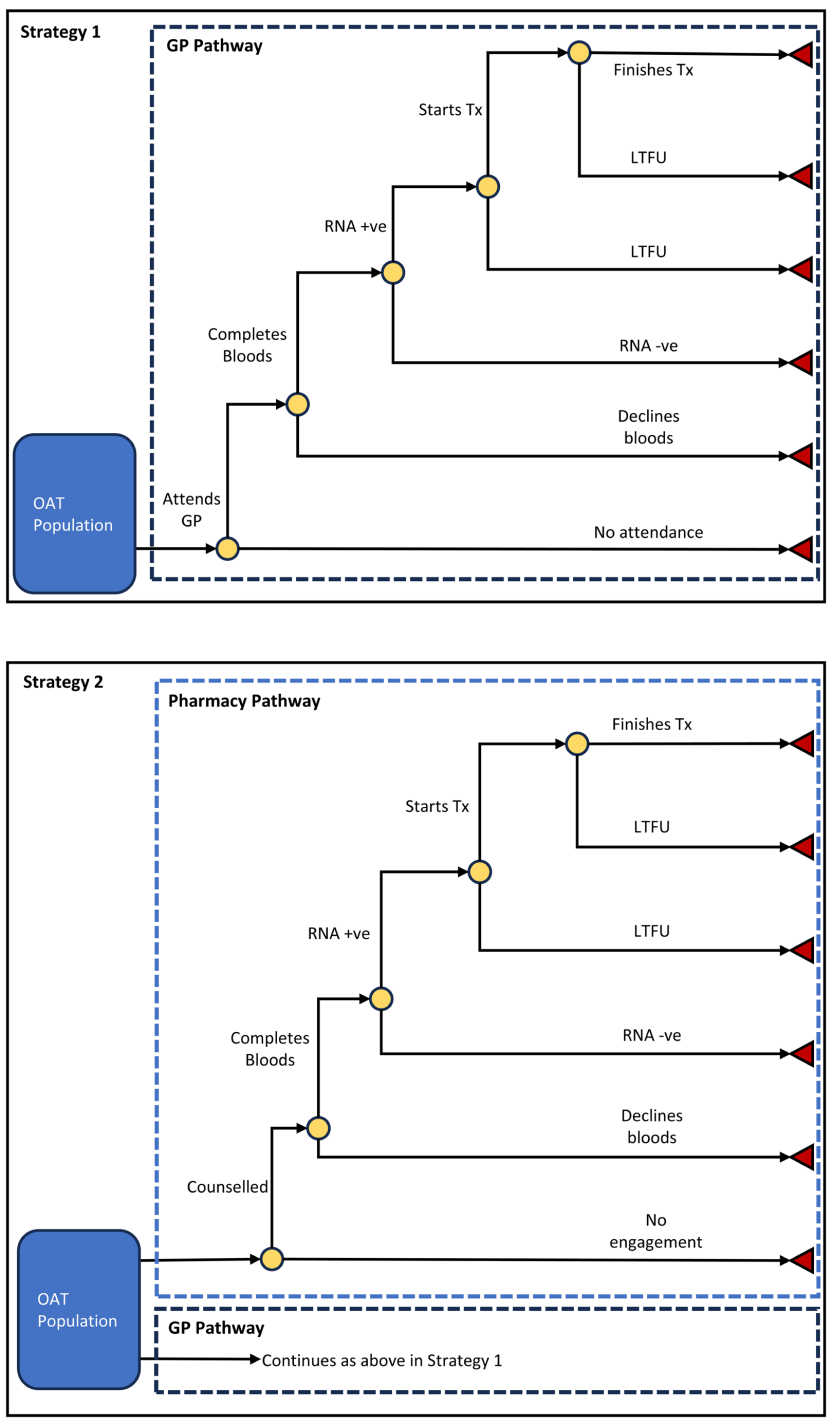
Diagram of model structure. Simplified diagram of decision analytic model. In Strategy 1, individuals on OAT can receive testing and treatment through the GP Pathway. In Strategy 2, the population can either flow through the GP Pathway or the Pharmacy Pathway. LTFU, Lost to follow up; Tx, Treatment; ‘+ve’, Positive; ‘‐ve’, Negative.

The decision analytic model was developed using Microsoft Excel for Microsoft 365 MSO, based on the clinical pathways described in REACH‐HCV trial [12], and consultation with clinical experts. The model, represented in Figure 1, begins with the annual cohort of individuals receiving OAT through community pharmacies in Australia. Two strategies were compared, the first (Strategy 1) where individuals on OAT can only be tested and treated for HCV through a GP or, the second (Strategy 2), where individuals could receive either testing and treatment through a GP or through the additional outreach programme in a pharmacy setting. Where individuals are receiving testing and treatment through the GP pathway in the model, they progress through the treatment cascade based on their probability of being tested for HCV, testing positive, accepting treatment if testing positive and finishing treatment if treatment is commenced. The GP treatment cascade in both strategies assumes that HCV pathology has been ordered by their doctor in an efficient manner, as an HCV antibody initially, with reflexive RNA testing and other necessary bloods if the serology is reactive. In Strategy 2, as in REACH‐HCV, individuals engaged through the pharmacy outreach programme move through the cascade of care from accepting counselling in their pharmacy, through to testing, treatment and potentially cure. In the original REACH‐HCV cascade, the prescription of DAAs was provided by a single physician. This is not a scalable plan, so our model instead assumes that treatment is prescribed via telehealth or in‐person consultation with a local, experienced general practitioner. In both care pathways of the model, all HCV‐diagnosed participants are assessed for cirrhosis by measuring the aspartate aminotransferase‐to‐platelet ratio index (APRI). We assumed that 5% of HCV‐diagnosed participants would then require an elastography scan as part of their pre‐treatment investigations based on their APRI results. This assumption is tested in the sensitivity analyses. All treatment regimens were assumed to be either 12‐week (base case) or 8‐week (sensitivity analysis) courses of government‐subsidised pangenotypic medication. Individuals in the model who finish the course of DAAs are assumed to be cured and are not required to re‐attend for further SVR testing. Side effects from DAAs were not considered in the model. The pharmacy outreach programme was assumed to be complimentary and not impact the expected number of tests, diagnoses and cures delivered in the GP pathway of Strategy 2, but this assumption was tested in sensitivity analyses.

## 4 Probabilities

A systematic literature search (Data S1) was undertaken to identify the key probabilities used in the model, in addition to the effectiveness data taken from the REACH‐HCV trial. A total of 156 relevant articles were identified in the search and reviewed at the title and abstract level by one author (JFG). From these articles, 56 were screened at the full‐text level by one author (JFG) and then discussed with the model development team (NS, MH, JD) to select appropriate values and ranges for use in the model. Model inputs are shown in Table 1. In Strategies 1 and 2, the GP care pathway begins with the assumption that all OAT clients continue their OAT prescription and therefore at some point in the 12‐month period must see a general practitioner to secure a repeat prescription. Based on recent literature specific to the Australian setting and for the population of either people on OAT, or PWID [14, 15, 16, 17], there are then probabilities for: OAT clients being tested for HCV by their GP over a 12‐month period; returning reactive serology if tested; returning a positive RNA result; commencing treatment if RNA is positive; finishing treatment once commenced. Strategy 2 additionally includes the probabilities of people on OAT accepting counselling in a pharmacy and being tested for HCV if they accepted counselling based on the REACH‐HCV trial [12]. The probability of returning reactive serology or a positive RNA result is identical for individuals in the model whether they receive testing through the GP or through the outreach programme [14, 15], and probabilities of commencing and completing treatment if RNA positive is based on proportions observed in the REACH‐HCV trial. Data from the intention to treat analysis from REACH‐HCV which included data from sites in the UK were used rather than only utilising data from the Australian sites, as this provided more conservative estimates of the effectiveness of the outreach programme.

**TABLE 1:** Parameter values used in the decision analytic model.

		DSA		PSA			
	Base case	LB	UB	Distribution	*α*	*β*	Source
GP pathway							
Probability of attending GP	1.000	1.000	1.000	N/A	N/A	N/A	Assumption
Probability of completing a test	0.1731	0.1385	0.2078	Beta	940	4489	Dawe et al. 2022 [14]
Probability that test is Ab positive	0.5604	0.4483	0.6725	Beta	524	411	Dawe et al. 2022 [14]
Probability that test is RNA positive	0.1471	0.1177	0.1766	Beta	123	713	Supp Table 2 of Valerio et al. 2022 [15]
Probability that patient commences treatment if tests positive on RNA	0.3785	0.3028	0.4543	Beta	120	197	Conway et al. 2022 [16]
Probability that patient finishes treatment if starts treatment	0.9589	0.7671	1.0000	Beta	1608	69	Yee et al. 2021 [17]
Pharmacy Pathway							
Probability of accepting counselling in pharmacy	0.5079	0.4063	0.6094	Beta	387	375	Byrne et al. 2022 [12]
Probability of being tested in pharmacy if accepts counselling	0.3721	0.2977	0.4465	Beta	144	243	Byrne et al. 2022 [12]
Probability that test is Ab positive	0.5604	0.4483	0.6725	Beta	524	411	Dawe et al. 2022 [14]
Probability that test is RNA positive	0.1471	0.1177	0.1766	Beta	123	713	Supp Table 2 of Valerio et al. 2022 [15]
Probability that patient commences treatment if tests positive on RNA	0.9565	0.7652	1.0000	Beta	22	1	Byrne et al. 2022 [12]
Probability that patient finishes treatment if starts treatment	0.8636	0.6909	1.0000	Beta	19	3	Byrne et al. 2022 [12]

*Note:* In the Probabilistic Sensitivity Analysis (PSA), beta distributions were assigned to all probabilities in the model, except the probability of attending the GP in the GP Pathway which was assumed as certain. Alpha and beta values for the beta distributions were based on the literature used for the base case estimates.

Abbreviations: Ab, antibody; DSA, deterministic sensitivity analysis; GP, general practitioner; LB, lower bound; PSA, probabilistic sensitivity analysis; UB, upper bound.

## 5 Costs

Resource utilisation for both the GP and outreach pathways was estimated using an ingredients‐based approach based on discussions with clinicians and staff involved in REACH‐HCV. Only costs incurred by the health system were analysed (Table 2). In the GP care pathway, this included the costs of attending the general practitioner for initial testing, the cost of investigations, the costs of returning to the practitioner if results were positive, medication and dispensing costs. Investigation costs included the consumable supplies required for venipuncture (Data S2), the cost of the initial serology and if a positive result was returned, the cost of the recommended workup for treatment. All costs in the GP care arm were incurred as variable expenses depending on the number of individuals in the model requiring those services.

**TABLE 2:** Variable cost parameters used in the decision analytic model.

Cost parameter	Deterministic analysis	PSA	Source
Pathology			
Hepatitis C serology	$15.65	Not varied	MBS #69475
Hep C PCR	$92.20	Not varied	MBS #69445 / 69499
Liver function tests (LFT)	$17.70	Not varied	MBS #66512
Full blood examination (FBE)	$16.95	Not varied	MBS #65070
Urea, electrolytes, creatinine (UEC)	$17.70	Not varied	MBS #66512
International Normalised Ratio (INR)	$13.70	Not varied	MBS #65120
HIV serology	$15.65	Not varied	MBS #69384
Hepatitis A + B serology	$29.25	Not varied	MBS #69478
Venepuncture	$6.93	Gamma, SD 10%	See Data S2
GP appointment			
GP consult type C	$76.95	Not varied	MBS#36
Pharmacy costs			
Government‐subsidised pangenotypic medication (per 1 month supply on general schedule, supplied by community pharmacy, 3 months treatment required)	$12,037.13	Not varied	PBS
Government‐subsidised pangenotypic medication. Alternative treatment used in sensitivity analysis (per 1 month supply on general schedule, supplied by community pharmacy, 2 months treatment required)	$17,895.47	Not varied	PBS
Dispensing fee (per 1 month supply)	$8.37	Not varied	PBS
Wholesale markup (per 1 month supply)	$54.14	Not varied	PBS
Administration, handling and infrastructure fee, tier 3 (per 1 month supply)	$99.62	Not varied	PBS
Elastography Scan (if required)			
Professional attendance fee	$81.05	Gamma, SD 10%	MBS #116
Scan billing (based on the cost of ultrasound)	$115.75	Gamma, SD 10%	MBS #55036

*Note:* In the PSA, costs with a set value in the MBS or PBS were not varied while costs obtained through quotes or estimates were varied using gamma distributions with a standard deviation equal to 10% of the mean. All costs in Australian Dollars (AUD), 2023.

Abbreviations: MBS, medicare benefits scheme; PBS, pharmaceutical benefits scheme; PCR, polymerase chain reaction; PSA, probabilistic sensitivity analysis; SD, standard deviation.

The outreach pathway included upfront project costs to establish the programme since this model of care is not standard (Data S3), in addition to variable costs for individuals requiring testing, workup and treatment. Initial expenditures for the intervention included the wages for the programme staff, staff benefits and on‐boarding costs, project overhead and administration costs, travel costs and any equipment for the nurses that could not be allocated on a per patient basis (e.g., torniquets, sharps disposal bin or cleaning supplies). In the base case of the intervention, 20 nurses are hired to visit the largest and busiest community pharmacies that provide OAT in Australia. There are more than 2700 pharmacies that dispense OAT in Australia [18]. We anticipated, based on discussion with outreach nurses from the REACH‐HCV trial, that with 20 field staff, we could expect over the course of the year to visit pharmacies caring for up to 65% of community OAT clients in Australia (24,127 individuals), aiming to at least have a discussion with half of these individuals. A population of this size would only require each nurse to deliver approximately 228 antibody tests over their approximate 250 workdays, based on the previously observed rate of success in engaging and testing in this population in trials [12]. This conservative estimate allows adequate consideration for the time it takes to engage a pharmacy site organise visitation days and travel to the location. These assumptions were tested in the one‐way sensitivity analyses. Services were valued using the Medicare Benefits Schedule (MBS), medications using the Pharmaceutical Benefits Scheme (PBS) and consumables from supplier quotes.

## 6 Analysis

The total number of tests, diagnoses, cures and costs were calculated for each strategy. Incremental cost‐effectiveness ratios were then calculated for the expected incremental cost incurred per additional individual with HCV cured using Strategy 2, compared to Strategy 1.

## 7 Uncertainty and Sensitivity Analysis

A probabilistic multivariate uncertainty analysis was undertaken to generate 95% uncertainty intervals for model outputs. Simulations were run with 500 parameter sets randomly sampled from model input uncertainties (Tables 1 and 2). These simulations were used to estimate the probability that the intervention would be considered cost‐effective for different willingness‐to‐pay thresholds.

One‐way sensitivity analyses (OWSA) were performed to identify model sensitivities to individual parameters. Base parameters were individually varied by +/− 20% to give upper and lower bound estimates. Key assumptions were also varied, such as the number of required nurses, the proportion of individuals requiring elastography and the treatment medication chosen.

Costs that have a set value such as those publicly listed in the MBS and PBS were not varied in the uncertainty or sensitivity analyses, while costs that were provided as quotes or estimates were all varied in the uncertainty analyses. The base case, uncertainty and sensitivity analyses were also estimated with the costs of medication and pharmacy dispensing excluded.

As this study used existing collections of published non‐identifiable data, no ethics committee approval was required.

## 8 Results

In the base case, for a hypothetically reachable population of 24,127 OAT clients in community pharmacies (two‐thirds of the total in Australia), we anticipated that 4178 individuals would complete HCV serology, 615 individuals with HCV would be identified (RNA positive) and 223 individuals would complete treatment in the GP pathway. This would incur costs to the health system of $10,965,634, at an average cost of $49,173 per cure (Table 3). Adding the outreach programme pathway to routine care (Strategy 2) resulted in a total of 8737 tests being delivered, 1285 individuals with HCV being identified and 777 completing treatment. This would incur $27,134,378 to the health system. The incremental cost per additional HCV cure gained by adding the outreach programme is $48,964 (Table 4) and the average cost per cure for Strategy 2 was $49,018.

**TABLE 3:** Base case results.

Result category	Strategy	Finding
Total costs	Strategy 1	$10,965,634
	Strategy 2	$38,100,012
Number of Ab tests completed	Strategy 1	4178
	Strategy 2	8737
Number of cases identified	Strategy 1	615
	Strategy 2	1285
Number completing treatment	Strategy 1	223
	Strategy 2	777
Average cost per case identified	Strategy 1	$17,841
	Strategy 2	$29,639
Average cost per case completing treatment	Strategy 1	$49,152
	Strategy 2	$49,018
Average cost per case completing treatment excluding DAA and dispensing costs	Strategy 1	$10,984
	Strategy 2	$7850

*Note:* Results from the base case analysis comparing Strategy 1, where OAT clients can utilise the GP Pathway and Strategy 2, where OAT clients can access either the GP Pathway or Pharmacy Pathway. All costs are AUD 2023.

Abbreviations: Ab, antibody; DAA, direct‐acting antivirals; ICER, incremental cost‐effectiveness ratio.

**TABLE 4:** Incremental costs and effects from the base case analysis.

	Total cost	Total cures	Incremental cost	Incremental cures	ICER
Strategy 1	$10,965,634	223	**—**	**—**	**—**
Strategy 2	$38,100,012	777	$27,134,402	554	$48,964

*Note:* Incremental results from the base case analysis comparing Strategy 1, where OAT clients can utilise the GP Pathway and Strategy 2, where OAT clients can access either the GP Pathway or Pharmacy Pathway. All costs are AUD 2023.

Abbreviation: ICER, incremental cost‐effectiveness ratio.

Most costs in both strategies were incurred at the stage of the model when individuals were prescribed and dispensed DAAs. In Strategy 2, medication and dispensing costs accounted for 86.5% of costs to the health system, while in Strategy 1 this was 77.81%. Without DAA and dispensing costs, the ICER in the base case was $6588, while the average cost per cure in the GP pathway alone was $10,984.

**FIGURE 2:**
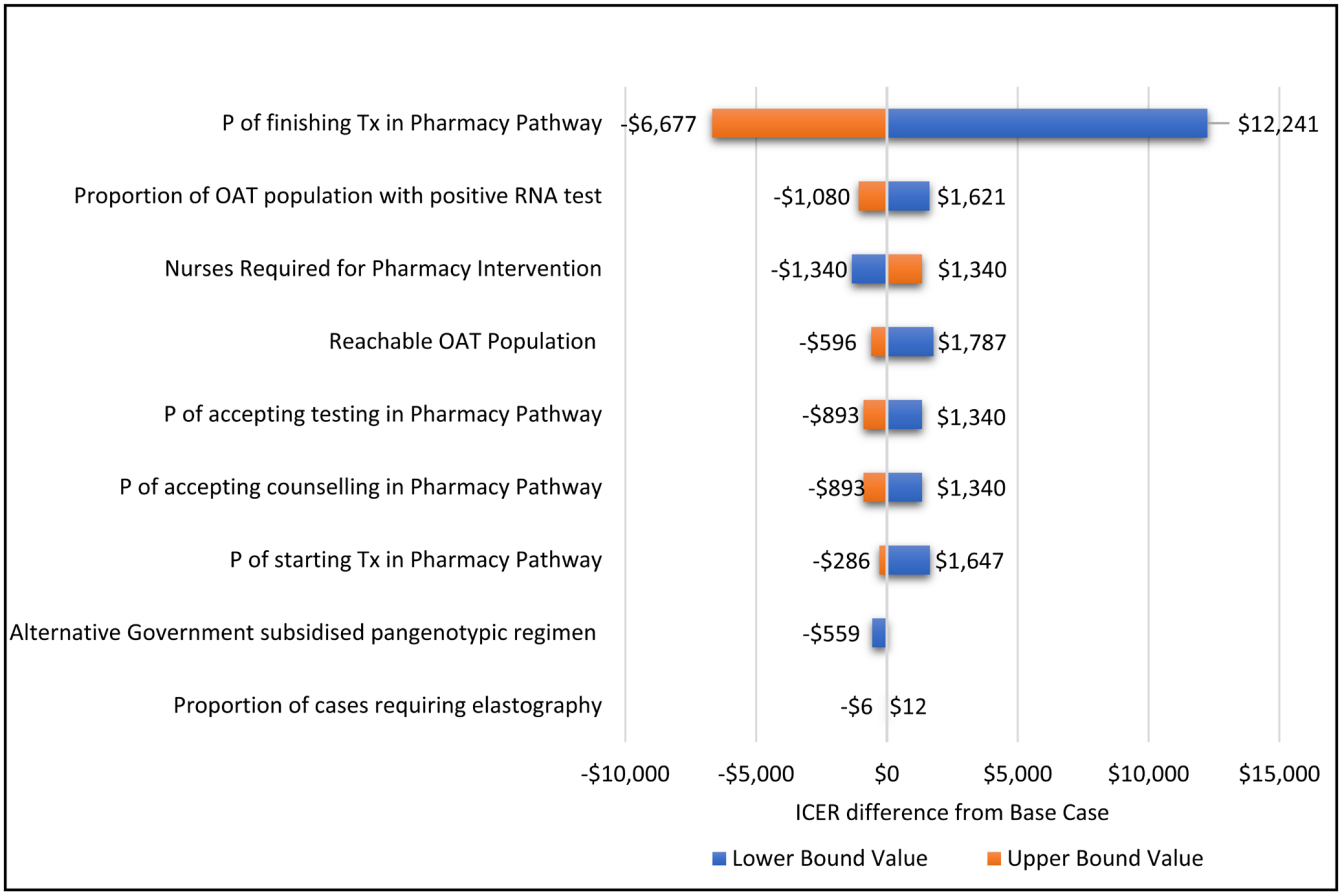
Tornado diagram of one‐way sensitivity analysis. The diagram shows the impact of 9 parameters on the Incremental Cost‐effectiveness Ratio (ICER). The ICER is the incremental cost incurred by the health system per incremental HCV cure gained in Strategy 2 compared to Strategy 1. All costs are AUD 2023. ICER, Incremental Cost‐effectiveness Ratio; OAT, Opioid agonist therapy; P, Probability; Tx, Treatment.

**FIGURE 3:**
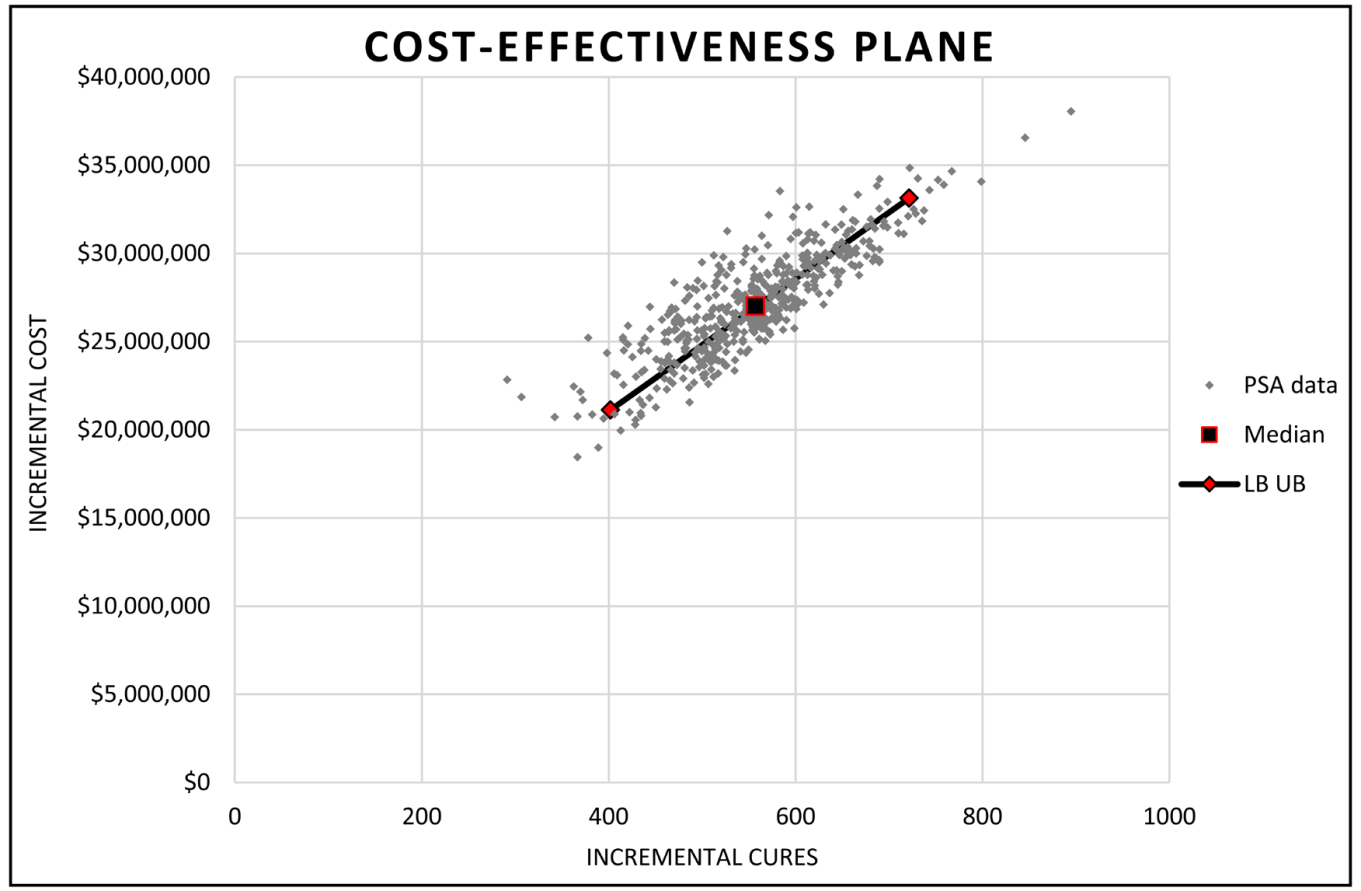
Incremental cost‐effectiveness scatter plot showing incremental cost and benefit of adding the pharmacy outreach intervention to GP care (Strategy 2) versus GP care alone (Strategy 1). Each grey diamond represents the incremental costs and cures estimated in a single iteration of the Monte Carlo simulation. All costs are AUD 2023, from the perspective of the health system. GP, General practice; PSA, Probabilistic sensitivity analysis; LB, Lower bound of Uncertainty Interval; UB, Upper bound of Uncertainty Interval.

**FIGURE 4:**
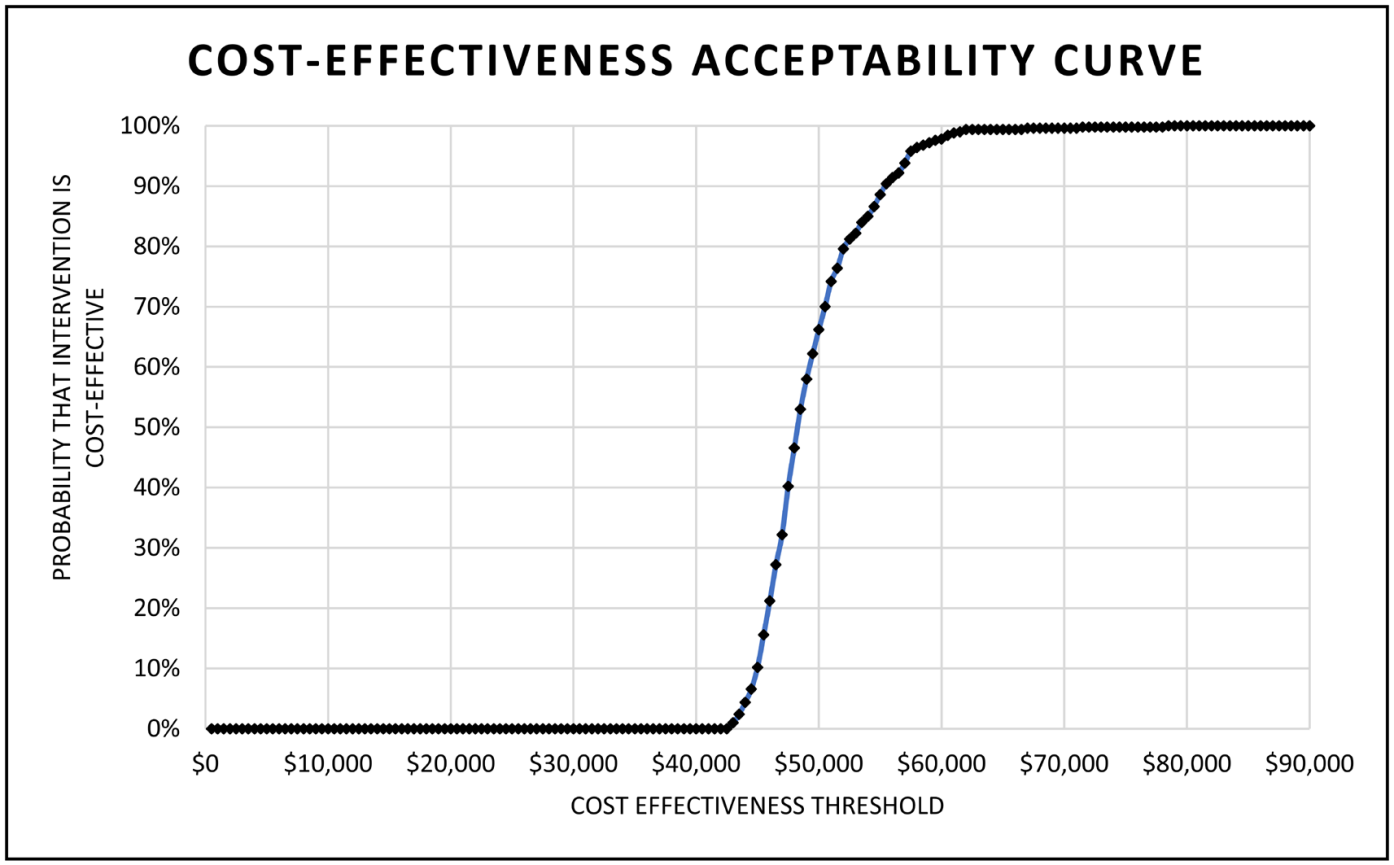
Cost‐effectiveness acceptability curve of adding the pharmacy outreach intervention to GP care (Strategy 2) versus GP care alone (Strategy 1). The curve shows the proportion of Monte Carlo iterations where the ICER of adding the pharmacy outreach intervention to the GP Pathway (Strategy 2) compared to the GP Pathway alone (Strategy 1) was below the cost‐effectiveness thresholds shown on the *x*‐axis. All costs are AUD 2023. GP: General practice.

The incremental cost per cure was most sensitive to changes in the probability that individuals who start treatment in the pharmacy pathway, also finish treatment (Data S4 & Figure 2). Decreasing this probability by 20% to the lower bound estimate (69.1%) resulted in a 25% increase in the estimated ICER to $61,205, while increasing the probability by 12% to the upper bound estimate (100%) resulted in a 13.64% reduction in the ICER to $42,287. Varying the number of nurses required for the programme, the expected reachable population size and the number requiring elastography did not significantly alter the observed ICER (Figure 2). In the probabilistic sensitivity analysis (PSA), the median ICER observed was $48,214. Meaning that, over the course of 500 iterations, the median incremental cost incurred by the health system was $48,214 per each additional cure Strategy 2 delivered compared to Strategy 1. Of all the observed ICER values, 95% fell between the range of $43,542 and $59,185. All iterations from the PSA are plotted in Figure 3 and the probability of Strategy 2 being cost‐effective at designated thresholds is shown in Figure 4.

As the base case assumed that adding the outreach programme in Strategy 2 would not diminish the number of tests, diagnoses and treatments delivered through the GP care pathway, this was tested in a scenario analysis (Data S5). In this analysis, introducing the outreach programme was assumed to result in diminished demand for routine care services in Strategy 2 (10%–30% reduction in tests) and was compared to the results achieved in Strategy 1. The ICER was not significantly altered compared to the base case in these analyses.

Given the high proportional cost of medications in the analysis, the OWSA and PSA were repeated excluding the pharmacy dispensing and medication costs (Data S6–S7B). After excluding these costs, the single parameter change that had the largest impact on the ICER was the proportion of OAT clients who test positive for HCV (Data S6). In the PSA, the median observed ICER was $6575 (UI $4928–$8969) (Data S7A).

## 9 Discussion

This analysis found that adding active HCV case finding and the provision of treatment through a community pharmacy‐based programme costs a similar amount per cure achieved to usual care through general practice alone while greatly increasing the number of tests and treatments delivered. Although there are upfront costs associated with the pharmacy intervention, the lower rates of loss to follow‐up in this treatment cascade result in a similar total cost per cure delivered between the two pathways. Starting treatment incurs the largest costs in the cascade of care, with a course of DAA treatment costing more than $36,000. If an individual is lost to follow‐up after commencing treatment, then a beneficial health outcome is not delivered despite these costs already being incurred. Therefore, the largest degree of variation in the ICER was seen when altering how effectively this outreach programme would maintain engagement with patients after they started treatment.

To our knowledge, this is the first cost‐effectiveness assessment of testing and treating HCV through community pharmacies in the Australian setting. Other assessments in Australia have included economic analyses comparing treatment through tertiary centres versus through primary care [19], tertiary care versus shared primary plus tertiary care [20], and care in prisons versus either primary or tertiary care [21]. In South Australia, an additional study compared the cost‐effectiveness of four models of treatment including specialist physician care, shared care between a physician and nurse, a specialist nurse alone (with specialist oversight) or through general practitioners [22]. These models were not comparable with our analysis, as they focussed on selected steps in the testing and treatment cascade. Either they analysed the costs of identifying individuals with HCV and completing pre‐treatment assessments [21] or they assessed the cost‐effectiveness of treating an individual after the diagnosis was already completed [19, 20, 22]. Acknowledging this heterogeneity, the models of care with more flexibility and accessibility generally resulted in higher levels of engagement, lower levels of loss to follow up and in many cases comparable or favourable cost consequences.

Outside of Australia, our analysis is comparable to previously published modelling of a very similar pharmacy‐based intervention in the UK setting [23]. In this study, each additional cure delivered by the programme incurred £39,094 (GBP 2019) from the perspective of the health system. This model of care differed slightly in that the pharmacists were trained to provide blood spot testing and oversee treatment, rather than serving as a source of referrals for outreach nurses.

There is no known willingness to pay a threshold per individual with HCV cured and no threshold was artificially set in this analysis, but it is presumably at least as much as the current costs being incurred per cure. The advantage of adding pharmacy‐based HCV services to the existing pathways in general practice is that the HCV burden is reduced more rapidly, preventing new infections. Other models, beyond the scope of this paper, have demonstrated that more rapid scale‐up of HCV services ultimately can lead to greater public health and economic benefits [24, 25].

The higher rate of retention in the pharmacy intervention arm is critically important in the cost‐effectiveness of this model. Previous studies, which have also demonstrated high client retention through pharmacy interventions, have postulated that providing testing and treatment onsite overcomes barriers such as the cost of travel and the difficulty of navigating health services [26, 27]. In addition, the development of positive long‐term relationships between pharmacy staff and OAT clients has been identified as a potential enabler of testing and treatment [26, 27]. A discrete choice experiment published in 2019 supported the importance of convenience, with individuals on OAT preferring to have testing in their own pharmacy compared to travelling to their GP [28]. Importantly, however, being treated with dignity and respect was valued higher than all other potential incentives to test, including financial [28].

There are limitations to this model. Firstly, this model considered only two possible treatment strategies for our population of interest and did not compare the potential cost‐effectiveness of all available models of care. Secondly, the results are only as accurate as the parameters used in the model. While many of the studies used to inform the model are based on multi‐state cohort studies, one study was only based on observations from Victoria [14], and therefore may be less representative of the national situation. Thirdly, the model adopts only a short‐term time horizon and therefore does not provide an estimate of the future costs potentially saved by increasing the number of cures delivered in the short term. However, the treatment of chronic HCV has already been shown to be cost‐effective with DAAs, even when the cost of medication was varied up to $100,000 per treatment course [29]. Fourthly, the model ends at the completion of treatment and does not require participants to re‐attend for SVR testing which is not a perfect representation of the current treatment guidelines in Australia. Our simplified model excluded this step as the observed rates of SVR testing were uncharacteristically low in the routine care arm of the REACH‐HCV trial. Including this step would have artificially made the outreach intervention seem considerably more cost‐effective at achieving cure and we know that 95% of individuals that complete a course of DAAs will achieve cure. Fifthly, the model assumes that there will be adequate staff to hire. HCV nurses are a small and specialised workforce and therefore being able to hire nurse specialists for this programme may prove to be difficult. A programme such as this would likely need to hire a mix of experienced HCV nurses and provide training to others willing to gain the required skills. Commencing the programme with a smaller team of nurses of 1–2 per jurisdiction and expanding through training as needed according to demand and uptake would be one way of ensuring staff costs do not exceed those modelled in this study. Alternatively, integrating this programme into pre‐existing care models in pharmacies, such as those that provide screening for hypertension and diabetes, could be another way to limit the impact of staffing limitations and associated costs.

The final limitation in this analysis was the choice to model to the intermediary outcome of HCV cure instead of a final endpoint such as quality‐adjusted life year (QALY) gained. Completing this analysis using a final endpoint such as QALYs would have increased the ease of comparison of this intervention and other public health projects. However, health state utility values for PWID and people on OAT are complicated by their concurrent socio‐economic challenges and the health benefits of cure can be extremely difficult to quantify in terms of quality‐of‐life scores [30, 31].

In Australia, pathology can only be requested and DAAs are prescribed by doctors and nurse practitioners. One practical challenge of modelling the impact of new service pathways is accounting for potential future regulatory changes. If pharmacists were able to order pathology and prescribe HCV treatments this could potentially change the relevance of this analysis. Realistically, however, community pharmacists already have several competing priorities and the addition of an outreach nurse to assist with counselling, phlebotomy and follow‐up is likely to be helpful regardless of who is prescribing the medication.

As we move towards elimination and prevalence continues to fall, identifying individuals with HCV, undertaking testing and completing treatment is expected to become increasingly difficult. To successfully meet national and international treatment targets we must increase testing, engagement and retention in treatment. Providing alternative pathways for accessing testing and treatment in safe environments with known engagement with at‐risk people, such as in community pharmacies that provide OAT, is one pathway forward. Our analysis has shown that despite the upfront costs of active case finding in this setting, the pathway incurs costs similar to those incurred through passive testing and treatment conducted in primary healthcare.

## Conflicts of Interest

A.R. reports Research Grants from Merck Sharpe and Dohme, Camurus, all unrelated to the submitted work. J.F.D. reports research grants and honoraria from Gilead, Abbvie and MSD, unrelated to the submitted work. M.H. has received investigator‐initiated research funding from Gilead Sciences and AbbVie to their institution; J.D. has received research funding from AbbVie and Gilead Sciences. The remaining authors have no conflicts to report.

## Supporting information


Data S1.

## Data Availability

The data that support the findings of this study are available from the corresponding author upon reasonable request.
