# Supplementary material for: Cost‐Effectiveness of Treating Hepatitis C in Clients on Opioid Agonist Therapy in Community Pharmacies Compared to Primary Healthcare in Australia
Source: J Viral Hepat. 2024 Oct 23;32(4):e14015. doi: 10.1111/jvh.14015 (PMC11897583; doi:10.1111/jvh.14015)
Supplement: Supplementary file 1 — Data S1. [file JVH-32-0-s001.docx]

**Supplementary Material**

**S1: Literature Search**

Search completed 11^th^ August 2023 on OVID Medline 1946 to August Week 2 2023

| 1 | Buprenorphine/ | 7156 |
| --- | --- | --- |
| 2 | Methadone/ | 13582 |
| 3 | Opioid Substitution Treatment.mp. | 361 |
| 4 | OST.mp. | 1890 |
| 5 | opioid agonist therapy.mp. | 470 |
| 6 | OAT.mp. | 8161 |
| 7 | Opioid replacement therapy.mp. | 109 |
| 8 | Opioid treatment program.mp. | 159 |
| 9 | "injecting drug use".mp. | 1763 |
| 10 | Drug Users/ | 4087 |
| 11 | people who inject drugs.mp. | 3122 |
| 12 | PWID.mp. | 2308 |
| 13 | Buprenorphine.mp. | 9186 |
| 14 | Methadone.mp. | 17389 |
| 15 | 1 or 2 or 3 or 4 or 5 or 6 or 7 or 8 or 9 or 10 or 11 or 12 or 13 or 14 | 40774 |
| 16 | hepatitis c/ or hepatitis c, chronic/ | 70798 |
| 17 | hepatitis C.mp. | 91252 |
| 18 | hepatitis c virus.mp. | 54236 |
| 19 | hepatitis C treatment.mp. | 1151 |
| 20 | hepatitis c virus infection.mp. | 8624 |
| 21 | HCV.mp. | 57680 |
| 22 | Hepatitis c diagnosis.mp. | 95 |
| 23 | Hepatitis c screening.mp. | 268 |
| 24 | 16 or 17 or 18 or 19 or 20 or 21 or 22 or 23 | 95726 |
| 25 | australia/ or australian capital territory/ or new south wales/ or northern territory/ or queensland/ or south australia/ or tasmania/ or victoria/ or western australia/ | 170552 |
| 26 | 15 and 24 and 25 | 318 |

- Results limited to years 2015 –2023 Inclusive: 156 studies
- 156 studies reviewed at title and abstract level
- 56 Studies screened at full text level

**S2: Consumable supplies used in venipuncture cost calculation.**

| **Consumable** | **Items per box** | **Cost per box** | **Cost per unit** | **Units required for venipuncture** | **Total** |
| --- | --- | --- | --- | --- | --- |
| Butterfly Vacutainer Needle | 50 | $95.14 | $1.90 | 1 | $1.90 |
| Nitrile Blue Gloves | 100 | $6.06 | $0.06 | 2 | $0.12 |
| Blood tube EDTA Purple 3ml | 100 | $29.33 | $0.29 | 1 | $0.29 |
| Blood tube Gold top SST 8.5ml plain tube | 100 | $46.85 | $0.47 | 3 | $1.41 |
| Blood tube Blue top CIT PLH 13X75 BLBL | 100 | $35.20 | $0.35 | 1 | $0.35 |
| Barrel tube needle holder 13&16MM | 250 | $66.19 | $0.26 | 1 | $0.26 |
| Livingstone biohazard specimen bag | 2000 | $163.35 | $0.08 | 1 | $0.08 |
| Injection tray compostable, white | 500 | $127.05 | $0.25 | 1 | $0.25 |
| Alcohol skin swab, Webcol | 200 | $3.90 | $0.02 | 1 | $0.02 |
| Swisper cotton balls | 60 | $4.40 | $0.07 | 1 | $0.07 |
| Bluey underpad sheet 60x400mm | 25 | $54.00 | $2.16 | 1 | $2.16 |
| **Total costs** |  |  |  |  | **$6.93** |

Consumables required as per outreach trial nurse on REACH-HCV trial. All costs sourced from private supplier quote provided to Burnet Institute. All costs above were varied in the probabilistic sensitivity analysis by assigning a gamma distribution, where the point estimate was the mean, and standard deviation was 10% of this mean. All costs in Australian Dollars (AUD), 2023.

**S3: Project costs (Pharmacy Pathway only) used in the decision analytic model.**

| **Cost Parameter** | **Deterministic Analysis** | **PSA** | **Source** |
| --- | --- | --- | --- |
| Wage (per nurse) | $92,305.20 | Gamma, SD 10% | Level 4, Grade 3 Nurse, Full Time, Nurses Award (MA000034)[1] |
| Nursing staff benefits and onboarding costs (per nurse) | $23,076.30 | Gamma, SD 10% | Assumption  (25% of wage) |
| Project Overhead and Administration (per nurse) | $23,076.30 | Gamma, SD 10% | Assumption  (25% of wage) |
| Staff transport (per nurse) | $10,000.00 | Gamma, SD 10% | Assumption |
| **Initial Equipment per Nurse** |  |  |  |
| Torniquet | $15.71 | Gamma, SD 10% | Supplier quote |
| Sharps disposal bin 5lt | $9.30 | Gamma, SD 10% | Supplier quote |
| Micropore Tape, bulk pack | $19.25 | Gamma, SD 10% | Supplier quote |
| Liv-wipes 70% alcohol wipes – 3 x 75 pack | $22.95 | Gamma, SD 10% | Supplier quote |

All costs in Australian Dollars (AUD), 2023. PSA: probabilistic sensitivity analysis. SD: Standard deviation. Consumable costs sourced from private supplier quote provided to Burnet Institute.

**S4: One-Way Sensitivity Analysis Results**

| **Sensitivity Analysis** | **Strategy 1 Cost** | **Strategy 2 Cost** | **Strategy 1 Cures** | **Strategy 2 Cures** | **Incremental Cost** | **Incremental Cures** | **ICER** |
| --- | --- | --- | --- | --- | --- | --- | --- |
| Base Case | $10,965,634 | $38,100,012 | 223 | 777 | $27,134,402 | 554 | $48,964 |
| P of completing a blood test in GPP 13.85% (LB) | $9,143,827 | $36,278,204 | 178 | 733 | $27,134,378 | 554 | $48,964 |
| P of completing a blood test in GPP 20.77% (UB) | $12,787,442 | $39,921,820 | 268 | 822 | $27,134,378 | 554 | $48,964 |
| P of RNA+ve is 11.78% (LB) | $9,257,823 | $31,683,854 | 178 | 622 | $22,426,031 | 443 | 50,585 |
| P of RNA+ve is 17.66% (UB) | $12,673,468 | $44,516,241 | 268 | 933 | $31,842,772 | 665 | 47,884 |
| P of starting Tx in GPP given RNA+ve is 30.28% (LB) | $9,259,023 | $36,393,401 | 178 | 733 | $27,134,378 | 554 | 48,964 |
| P of starting Tx in GPP given RNA+ve is 45.43% (UB) | $12,672,246 | $39,806,623 | 268 | 822 | $27,134,378 | 554 | 48,964 |
| P of finishing Tx in GPP given starts Tx is 76.71% (LB) | $10,965,634 | $38,100,012 | 178 | 733 | $27,134,378 | 554 | 48,964 |
| P of finishing Tx in GPP given starts Tx is 100% (UB) | $10,965,634 | $38,100,012 | 233 | 787 | $27,134,378 | 554 | 48,964 |
| P of accepting counselling in PP 40.63% (LB) | $10,965,634 | $33,267,237 | 223 | 666 | $22,301,602 | 443 | 50,304 |
| P of accepting counselling in PP 60.95% (UB) | $10,965,634 | $42,932,787 | 223 | 888 | $31,967,153 | 665 | 48,071 |
| P of accepting testing PP given counselled is 29.77% (LB) | $10,965,634 | $33,267,237 | 223 | 666 | $22,301,602 | 443 | 50,304 |
| P of accepting testing in PP given counselled is 44.65% (UB) | $10,965,634 | $42,932,787 | 223 | 888 | $31,967,153 | 665 | 48,071 |
| P of starting Tx in PP given RNA +ve is 76.52% (LB) | $10,965,634 | $33,403,290 | 223 | 666 | $22,437,656 | 443 | 50,611 |
| P of starting Tx in PP given RNA +ve is 100% (UB) | $10,965,634 | $39,167,449 | 223 | 802 | $28,201,814 | 579 | 48,678 |
| P of finishing Tx given starts Tx in PP is 69.09% (LB) | $10,965,634 | $38,100,012 | 223 | 666 | $27,134,378 | 443 | 61,205 |
| P of finishing Tx given starts Tx in PP is 100% (UB) | $10,965,634 | $38,100,012 | 223 | 865 | $27,134,378 | 642 | 42,287 |
| Number of PP nurses required is 15 (LB) | $10,965,634 | $37,357,387 | 223 | 777 | $26,391,753 | 554 | 47,624 |
| Number of PP nurses required is 25 (UB) | $10,965,634 | $38,842,637 | 223 | 777 | $27,877,003 | 554 | 50,304 |
| Cases requiring elastography scan is 2.5% (LB) | $10,962,610 | $38,093,687 | 223 | 777 | $27,131,077 | 554 | 48,958 |
| Cases requiring elastography scan is 10% (UB) | $10,971,682 | $38,112,661 | 223 | 777 | $27,140,979 | 554 | 48,976 |
| Reachable OAT Population is smaller (50% community pharmacy OAT clients) | $8,224,234 | $29,317,660 | 167 | 583 | $21,093,426 | 416 | 50,751 |
| Reachable OAT Population is larger (75% community pharmacy OAT clients) | $12,336,351 | $42,491,240 | 251 | 874 | $30,154,889 | 623 | 48,369 |
| Alternative Government subsidised pangenotypic medication (2-month course in place of 3-month course) | $10,853,353 | $37,678,075 | 223 | 777 | $26,824,722 | 554 | 48,405 |

Results from the one-way sensitivity analysis. Incremental costs and cures were calculated comparing Strategy 2 to Strategy 1. All costs are AUD 2023. ICER: Incremental Cost-effectiveness Ratio. GPP: GP Pathway. LB: lower bound. OAT: Opioid agonist therapy. P: Probability. PP: Pharmacy Pathway. RNA+ve: RNA positive. Tx: Treatment. UB: Upper bound.

**S5: Results from scenario analysis where the introduction of the Pharmacy Pathway diminishes testing in the GP Pathway in Strategy 2**

|  | **Strategy 1 Costs** | **Strategy 2 Costs** | **Strategy 1 Cures** | **Strategy 2 Cures** | **Incremental Cost** | **Incremental Cures** | **ICER** |
| --- | --- | --- | --- | --- | --- | --- | --- |
| PP introduction decreases GPP testing by 10% in Strategy 2 | $10,965,634 | $37,189,108 | 223 | 755 | $26,223,474 | 532 | $49,305 |
| PP introduction decreases GPP testing by 20% in Strategy 2 | $10,965,634 | $36,278,204 | 223 | 733 | $25,312,570 | 510 | $49,676 |
| PP introduction decreases GPP testing by 30% in Strategy 2 | $10,965,634 | $35,367,301 | 223 | 710 | $24,401,666 | 487 | $50,082 |

Incremental costs and cures were calculated comparing Strategy 2 to Strategy 1. All costs are AUD 2023. GPP: GP Pathway. ICER: Incremental Cost-effectiveness Ratio. PP: Pharmacy Pathway.

**S6: Tornado diagram of One-Way Sensitivity Analysis – analysis excluding medication costs**

This analysis excluded the cost of medication and pharmacy dispensing. The diagram shows the impact of 8 parameters on the Incremental Cost-effectiveness Ratio (ICER). The ICER is the incremental cost incurred by the health system per incremental HCV cure gained by adding the pharmacy outreach program (Strategy 2) compared to utilising only the GP Pathway (Strategy 1). All costs are AUD 2023. ICER: Incremental Cost-effectiveness Ratio. OAT: Opioid agonist therapy. P: Probability. Tx: Treatment.

**Figure 7A: Incremental cost-effectiveness scatter plot showing incremental cost and benefit of adding the pharmacy outreach intervention to GP care (Strategy 2) versus GP care alone (Strategy 1) - analysis excluding medication costs**

**S7A Legend:** This analysis excluded the cost of medication and pharmacy dispensing. Each grey diamond represents the incremental costs and cures estimated in a single iteration of the Monte Carlo simulation. All costs are AUD 2023, from the perspective of the health system. PSA: probabilistic sensitivity analysis. LB: lower bound of Uncertainty Interval. UB: Upper bound of Uncertainty Interval.

**Figure S7B: Cost-effectiveness acceptability curve of adding the pharmacy outreach intervention to GP care (Strategy 2) versus GP care alone (Strategy 1) - analysis excluding medication costs**

**S7B Legend:** This analysis excluded the cost of medication and pharmacy dispensing. The curve shows the proportion of Monte Carlo iterations where the ICER of adding the pharmacy outreach intervention to the GP Pathway (Strategy 2) compared to the GP Pathway alone (Strategy 1) was below the cost-effectiveness thresholds shown on the x axis. All costs are AUD 2023.

**S8: CHEERS 2022 Checklist**

| **Topic** | **No.** | **Item** | **Location where item is reported** |
| --- | --- | --- | --- |
| **Title** |  |  |  |
|  | 1 | Identify the study as an economic evaluation and specify the interventions being compared. | Title |
| **Abstract** |  |  |  |
|  | 2 | Provide a structured summary that highlights context, key methods, results, and alternative analyses. | Abstract |
| **Introduction** |  |  |  |
| **Background and objectives** | 3 | Give the context for the study, the study question, and its practical relevance for decision making in policy or practice. | Introduction, paragraphs 1-4 of manuscript |
| **Methods** |  |  |  |
| **Health economic analysis plan** | 4 | Indicate whether a health economic analysis plan was developed and where available. | No HEAP developed. |
| **Study population** | 5 | Describe characteristics of the study population (such as age range, demographics, socioeconomic, or clinical characteristics). | Study Design and Methods: Paragraph 1 |
| **Setting and location** | 6 | Provide relevant contextual information that may influence findings. | Study Design and Methods: Paragraph 1 |
| **Comparators** | 7 | Describe the interventions or strategies being compared and why chosen. | Study Design and Methods: Paragraph 2 |
| **Perspective** | 8 | State the perspective(s) adopted by the study and why chosen. | Study Design and Methods: Paragraph 2 |
| **Time horizon** | 9 | State the time horizon for the study and why appropriate. | Study Design and Methods: Paragraph 2 |
| **Discount rate** | 10 | Report the discount rate(s) and reason chosen. | Study Design and Methods: Paragraph 2 |
| **Selection of outcomes** | 11 | Describe what outcomes were used as the measure(s) of benefit(s) and harm(s). | Analysis: Paragraph 1 |
| **Measurement of outcomes** | 12 | Describe how outcomes used to capture benefit(s) and harm(s) were measured. | Study Design and Methods: Paragraph 2  &  Analysis: Paragraph 1 |
| **Valuation of outcomes** | 13 | Describe the population and methods used to measure and value outcomes. | Not Applicable |
| **Measurement and valuation of resources and costs** | 14 | Describe how costs were valued. | Costs: Paragraphs 1 and 2 |
| **Currency, price date, and conversion** | 15 | Report the dates of the estimated resource quantities and unit costs, plus the currency and year of conversion. | Study Design and Methods: Paragraph 2 |
| **Rationale and description of model** | 16 | If modelling is used, describe in detail and why used. Report if the model is publicly available and where it can be accessed. | Model Structure: Paragraph 1 |
| **Analytics and assumptions** | 17 | Describe any methods for analysing or statistically transforming data, any extrapolation methods, and approaches for validating any model used. | Uncertainty and Sensitivity Analysis: Paragraphs 1-3 |
| **Characterising heterogeneity** | 18 | Describe any methods used for estimating how the results of the study vary for subgroups. | Not applicable |
| **Characterising distributional effects** | 19 | Describe how impacts are distributed across different individuals or adjustments made to reflect priority populations. | Not applicable |
| **Characterising uncertainty** | 20 | Describe methods to characterise any sources of uncertainty in the analysis. | Uncertainty and Sensitivity Analysis: Paragraphs 1-3 |
| **Approach to engagement with patients and others affected by the study** | 21 | Describe any approaches to engage patients or service recipients, the general public, communities, or stakeholders (such as clinicians or payers) in the design of the study. | Model Structure: Paragraph 1 |
| **Results** |  |  |  |
| **Study parameters** | 22 | Report all analytic inputs (such as values, ranges, references) including uncertainty or distributional assumptions. | Table 1, Table 2, S2, S3 |
| **Summary of main results** | 23 | Report the mean values for the main categories of costs and outcomes of interest and summarise them in the most appropriate overall measure. | Results Paragraph 1-2, Table 3, Table 4 |
| **Effect of uncertainty** | 24 | Describe how uncertainty about analytic judgments, inputs, or projections affect findings. Report the effect of choice of discount rate and time horizon, if applicable. | Results paragraph 3-5, Figure 2, Figure 3A, Figure 3B, S4, S5, S6, S7A, S7B |
| **Effect of engagement with patients and others affected by the study** | 25 | Report on any difference patient/service recipient, general public, community, or stakeholder involvement made to the approach or findings of the study | Not completed |
| **Discussion** |  |  |  |
| **Study findings, limitations, generalisability, and current knowledge** | 26 | Report key findings, limitations, ethical or equity considerations not captured, and how these could affect patients, policy, or practice. | Discussion paragraph 1-8 |
| **Other relevant information** |  |  |  |
| **Source of funding** | 27 | Describe how the study was funded and any role of the funder in the identification, design, conduct, and reporting of the analysis | Sources of Funding: Page 1 |
| **Conflicts of interest** | 28 | Report authors conflicts of interest according to journal or International Committee of Medical Journal Editors requirements. | Conflicts of Interest Statement:  Page 1 |

*From:* Husereau D, Drummond M, Augustovski F, et al. Consolidated Health Economic Evaluation Reporting Standards 2022 (CHEERS 2022) Explanation and Elaboration: A Report of the ISPOR CHEERS II Good Practices Task Force. Value Health 2022;25. <doi:10.1016/j.jval.2021.10.008>

**References**

1. Fair Work Ombudsman. Nurses Award 2020 MA000034. Australian Government, 2020.
